# Exploratory study on classification of diabetes mellitus through a combined Random Forest Classifier

**DOI:** 10.1186/s12911-021-01471-4

**Published:** 2021-03-20

**Authors:** Xuchun Wang, Mengmeng Zhai, Zeping Ren, Hao Ren, Meichen Li, Dichen Quan, Limin Chen, Lixia Qiu

**Affiliations:** 1 Department of Health Statistics, School of Public Health, Shanxi Medical University, Taiyuan, Shanxi China; 2 Shanxi Centre for Disease Control and Prevention, Taiyuan, 030012 Shanxi China; 3 Shanxi Provincial People’s Hospital, Taiyuan City, Shanxi Province China

**Keywords:** Diabetes mellitus, Classification, Random Forest Classifier, Imbalanced data, Indicators

## Abstract

**Background:**

Diabetes Mellitus (DM) has become the third chronic non-communicable disease that hits patients after tumors, cardiovascular and cerebrovascular diseases, and has become one of the major public health problems in the world. Therefore, it is of great importance to identify individuals at high risk for DM in order to establish prevention strategies for DM.

**Methods:**

Aiming at the problem of high-dimensional feature space and high feature redundancy of medical data, as well as the problem of data imbalance often faced. This study explored different supervised classifiers, combined with SVM-SMOTE and two feature dimensionality reduction methods (Logistic stepwise regression and LAASO) to classify the diabetes survey sample data with unbalanced categories and complex related factors. Analysis and discussion of the classification results of 4 supervised classifiers based on 4 data processing methods. Five indicators including Accuracy, Precision, Recall, F1-Score and AUC are selected as the key indicators to evaluate the performance of the classification model.

**Results:**

According to the result, Random Forest Classifier combining SVM-SMOTE resampling technology and LASSO feature screening method (Accuracy = 0.890, Precision = 0.869, Recall = 0.919, F1-Score = 0.893, AUC = 0.948) proved the best way to tell those at high risk of DM. Besides, the combined algorithm helps enhance the classification performance for prediction of high-risk people of DM. Also, age, region, heart rate, hypertension, hyperlipidemia and BMI are the top six most critical characteristic variables affecting diabetes.

**Conclusions:**

The Random Forest Classifier combining with SVM-SMOTE and LASSO feature reduction method perform best in identifying high-risk people of DM from individuals. And the combined method proposed in the study would be a good tool for early screening of DM.

**Supplementary Information:**

The online version contains supplementary material available at 10.1186/s12911-021-01471-4.

## Background

At present, the global prevalence of Diabetes mellitus (DM) shows a rapid upward trend, and the number of diabetic patients on the increase. DM has become the third chronic non-communicable disease that hits patients after tumors, cardiovascular and cerebrovascular diseases, and has become one of the major public health problems in the world [1, 2]. In China, amid the booming economy, improving living standards and the ageing society, the accelerating pace of life and the phenomenon of aging society, the prevalence of DM is also mounting year by year [3–6]. Recent studies have shown that improving lifestyle and medication interventions can prevent diabetic complications, and it may help prevent the onset of type 2 diabetes mellitus (T2DM) [7–11]. Before the onset of diabetes, interventions to avoid diabetes or to delay its occurrence turns to be the most effective and economical way to control type 2 diabetes mellitus (T2DM). Therefore, it is important to identify individuals at high risk for T2DM and to establish prevention strategies for T2DM.

In recent years, machine learning such as big data analysis and data mining has attracted public attention, especially in the field of medical and health, data mining is widely used and plays an irreplaceable role [12–15]. Among them, machine learning and deep learning are increasingly employed for disease risk prediction, and considerable research performances have been achieved [16, 17]. For disease risk prediction, it mainly concerns classification and recognition technology in data mining. More common classification learning algorithms relate to Logistic Regression [18], Decision Trees [19], Neural Networks [20], Naive Bayes [21], and Support Vector Machines [22]. Considering the limited scope of application of each single classification algorithm, low generalization ability, high risk, and unstable classification performance, how to construct a model with strong generalization performance from the data structure attracts more and more researchers. The ensemble method came into being [23], which is different from the traditional learning method that only constructs one learner through the training set. The ensemble learning aims for a set of classifiers, using a certain combination for the output of each classifier and it helps to integrate the accuracy and differences of different classifiers to improve the generalization ability. It is worth noting that the computational expense to construct an ensemble is not much higher than that of a single classifier, as a result of which, ensemble learning is more widespread. In this study, two different ensemble classification algorithms will be used to construct a predictive model, and a comparative analysis will be made with the traditional single classification algorithm.

Data preprocessing refers to a series of operations before the classification task, including the collation of original data, extraction of feature vectors and class balance. In this study, feature engineering and class balance will be analyzed and discussed in detail, which are the most critical factors for prediction performance of the classification models. Models without variable screening not only have poor reproducibility in different medical environments, but also bring huge computational costs in operation and post-maintenance, and cannot be effectively applied to clinical practice. A large number of studies on predictive models have shown that variable screening can make the constructed model more concise, with less correlation between variables, and reduce the risk of overfitting by removing irrelevant features, thereby making the model performance better [24]. Besides, the imbalance problem is one of the top10 challenging problems in data mining [25]. It occurs in many real-world domains [26, 27], and will hurt the performance of the training model in the training phase. It usually tends to generate models that maximize the overall classification accuracy, and the minority class is usually ignored [28], so the recognition rate of the minority category is low [29–31]. However, the minority class usually represents a concept with greater interest than the majority class. Thus, they may be inaccurate for the class imbalance problems. Nowadays, resampling technology has been gradually applied to the processing of unbalanced data for its simplicity and its easy implementation [32–35].

In this article, we collected 4105 valid samples to analyze high-risk groups of diabetes. In consideration of the barriers of unbalanced proportion and information redundancy in data, a combined method based on the Random Forest algorithm was proposed for better classification performance. With the help of resampling technology and feature reduction methods, important diabetes-related factors can be accurately extracted and the performance of the diabetes classification model can be improved. Combining the above data processing methods, we constructed a total of four combined classification models of ensemble algorithms (Random Forest, Gradient Boosting) and single classification algorithms (Logistic Regression, Support Vector Machine). The results showed the potential of the combined model based on Random Forest ensemble algorithms to predict diabetes classification. The proposed combined method will be a powerful tool to provide auxiliary decision-making for early screening of diabetes.

## Methods

### Study participants

Participants were enrolled in the China National Chronic Disease Survey conducted in Shanxi Province in 2013. The survey adopted a multi-stage stratified random sampling method for a representative sample. Four towns (streets, groups) are randomly selected from 8 monitoring points in Datong Xian, Shuocheng Qu, Lin Xian, Xinghua Ling, Pingding Xian, Yuci Qu, Huguan Xian, Jiang Xian in Shanxi Province. These monitoring points are relatively evenly distributed in Shanxi Province. The specific sampling method and process are shown in Table S1 (Additional file 1: Table S1). 5000 people should be surveyed this time, and 4776 people were actually surveyed, with the response rate accounting for 95.52%. This study has been approved by the China Chronic Disease Center Ethics Committee (No. 201307). All study participants or their agents signed the informed consent. All experiments were carried out under relevant guidelines and regulations.

#### Survey content and method

(1) Questionnaire survey (Additional file 2: questionnaire): Before collecting the data, all participants received a written informed consent. After signing the informed consent form, all participants were asked to fill a chronic disease questionnaire developed by the Chinese Center for Disease Control and Prevention (CDC). Uniformly trained investigators conducted direct face-to-face questionnaire interviews. The questionnaire included the following information: general demographic characteristics (such as age, gender, region, occupation, and education level), lifestyles (such as eating habits, drinking, smoking, and physical activity) and past medical history (such as hyperlipidemia and hypertension). (2) Anthropometric measures: Body measurement mainly involves height, weight, waist circumference and blood pressure. When measuring height and weight, participants is are required to take off his shoes, hat and coat. The measuring tools are a height meter with an accuracy of 0.1 cm and an electronic scale with 0.1 g. Waist measurement uses a waist ruler with an accuracy of 0.1 cm. Repeat the measurement twice. After ensuring that the error of the two measurements is less than 2 cm, the second measurement shall prevail. The blood pressure was measured when participants are sitting and resting for 5 min. Third consecutive blood pressure (BP) readings were taken by an electronic sphygmomanometer (OMRON HEM-7071 or HEM-770A), with an accuracy of 1 mmHg; finally, take the average of the three blood pressure measurements. (3) Laboratory assays: Detection indicators include blood sugar, blood lipids, glycosylated hemoglobin, etc. The samples for blood glucose testing should be stored in refrigerator at 2 ~ 8℃ and sent to the local designated laboratory for testing within 48 h; other blood samples need to be stored at a low temperature of − 60 ℃ to − 80 ℃. In areas where there is no ultra-low temperature storage equipment, they should be stored at  ≤  − 20 ℃ and sent to the nationally designated medical inspection agency for unified determination within one month.

#### Definitions

- ① Diabetes Mellitus:refers to those with fasting blood glucose level ≥7mmol/L or two-hour postprandial blood glucose(2hPG)≥11.1mmol/L or those previously diagnosed with diabetes [36]. ② Hypertension: according to the diagnostic criteria of hypertension in the "Guidelines for the Prevention and Control of Hypertension in Chinese Residents": systolic blood pressure ≥140mmHg and/or diastolic blood pressure ≥90mmHg, or those who have been previously diagnosed with hypertension but have normal blood pressure after taking the drug [37]. ③ Dyslipidemia was defined according to Chinese Guidelines on Prevention and Treatment of Dyslipidemia in Adults published in 2007. Hyperlipidemia is defined as one or more of the following abnormal lipid characteristics: elevated concentration of total cholesterol (TC; ≥6.22mmol/L), lowdensity lipoprotein cholesterol (LDL-C; ≥4.14mmol/L), triglycerides (TG; ≥2.26mmol/L) or decreased level of high-density lipoprotein cholesterol (HDL-C; <1.04mmol/L) [38]. ④ Participants who smoked ≥1 cigarette a day in the past 6 months were defined as smokers. ⑤ Drinking refers to drinking alcohol at least 1 times a week, with an alcohol intake of 50 g or more for 6 consecutive months; ⑥ Body weight was categorized as low body weight (body mass index (BMI) <18.5kg/m2), normal weight (BMI:18.5 kg/m2 ~ 24 kg/m2), overweight (BMI:24 kg/m2 ~ 28 kg/m2), and obese (BMI≥28 kg/m2) [39].⑦ Central obesity refers to male waist circumference ≥85cm, female waist circumference ≥80cm [40]. ⑧ Heart rate was categorized into bradycardia (<60 beats/min), normal heart rate (60-100 beats/min) and tachycardia (>100 beats/min). ⑨ Physical activity is classified into insufficient physical activity, normal physical activity, and adequate physical activity according to the upper quartile and lower quartile of metabolic equivalents.

### Dataset

A total of 4776 people were surveyed in this study. After the data were sorted, 671 surveyors with missing data were removed, and the data were 4105 complete. Among them, there are 386 patients with diabetes with the imbalance ratio 9.53, which cause the class imbalance problem. Given this problem, the SVM-SMOTE algorithm was used to address the data. Since the detection of DM is the focus of attention, diabetic patients are classified as positive and non-diabetic are classified as negative. At the same time, according to respondents’ demographic information, lifestyle, eating habits, physiological status and other indicators, 18 variables are selected for each sample. The specific variable names and assignments are shown in Table 1 and Table S2 (Additional file 1: Table S2).

**Table 1:** Variables and their assignments

Factors	Assignment
Gender (${\mathrm{x}}_{1}$)	Male = 1*; Female = 2
Age (${\mathrm{x}}_{2}$)	< 40 = 1*;40 ~ = 2;60 ~ = 3;
Region (${\mathrm{x}}_{3}$)	Village = 1*; City = 2
Ethnic (${\mathrm{x}}_{4}$)	Ethnic Han = 1*; National minority = 2
Culture level (${\mathrm{x}}_{5}$)	Elementary school and below = 1*; Junior and senior high school = 2; College degree and above = 3
Occupation (${\mathrm{x}}_{6}$)	Farmer = 1*; Retirees or unemployers = 2; Employers = 3; other = 4
Marital status (${\mathrm{x}}_{7}$)	Spinsterhood = 1*; Married or cohabiting = 2; Divorced, widowed or separated = 3
Smoking (${\mathrm{x}}_{8}$)	NO = 0; YES = 1
Drinking status (${\mathrm{x}}_{9}$)	Never drink = 1*; Drinking every day = 2; Frequently = 3; Occasionally = 4
Physical activity (${\mathrm{x}}_{10}$)	Insufficient = 1*; Normal = 2; Sufficient = 3
Fresh fruit (${\mathrm{x}}_{11}$)	< 100 g/d = 1*;100 ~ 200 g/d = 2; > 200 g/d = 3
Vegetable intake level (${\mathrm{x}}_{12}$)	< 400 g/d = 1*;400 ~ 500 g/d = 2; > 500 g/d = 3
Meat (${\mathrm{x}}_{13}$)	< 50 g/d = 1*;50 ~ 100 g/d = 2; > 100 g/d = 3
Heart rate(${\mathrm{x}}_{14}$)	Bradycardia = 1; Normal = 2*; Tachycardia = 3
BMI(${\mathrm{x}}_{15}$)	< 18.5 = 1*;18.5 ~ = 2, 24.0 ~ = 3;28.0 ~ = 4
Central obesity (${\mathrm{x}}_{16}$)	NO = 0*; YES = 1
Hypertension (${\mathrm{x}}_{17}$)	NO = 0*; YES = 1
Hyperlipidemia(${\mathrm{x}}_{18}$)	NO = 0*; YES = 1
Diabetes mellitus (y)	NO = 0*; YES = 1

^*^^Reference standard^

#### Sampling working principle and process

We mainly employed resampling methods for unbalanced data. The resampling method can be roughly divided into two levels: data level and algorithm level. The data level includes over-sampling and under-sampling. The under-sampling methods eliminate the majority class instances while the over-sampling methods increase the minority class instances to obtain a desirable rate of class distribution. The algorithm level includes SMOTE, random over-sampling, etc., of which SMOTE was proposed by Chawla [35] in 2002. This method can effectively avoid the "over-fitting" problem [41]. However, the SMOTE algorithm has a certain degree of blindness in the process of new sample synthesis. It cannot accurately control the number of newly synthesized samples, nor can it make discriminating selections for minority samples. At the same time, in the process of synthesizing new samples, the information of most neighboring samples is not fully considered, which often leads to serious sample confusion and low classification accuracy. In view of the shortcomings of the SMOTE algorithm, scholars have proposed many improved algorithms. This study will use the SVM-SMOTE resampling technique proposed by Hien M. Nguyen et al. in 2011 [42]. In their method, the boundary line area is approximated by the support vectors obtained after training the standard SVM classifier on the original training set. Interpolation or extrapolation techniques will be used to randomly create new instances based on the density of the surrounding majority class instances along the line connecting each minority class support vector with its closest neighbor [42]. This study was implemented using the SVM-SMOTE statement in the “Implearn package” in Python software. In the SVM-SMOTE statement, we set the inter-class ratio after SVMSMOTE sampling to 1:1, thereby equalizing the unbalanced data set.

#### Feature dimensionality reduction methods

Variable screening is mainly to delete variables in the database that are not related to the outcome, and increase the signal-to-noise ratio in the database to improve the generalization ability of the model. For commonly used screening methods, they could be mainly divided into two categories; one is based on traditional regression, and the other on decision tree model [43]. Two methods were selected in this study: the stepwise logistic regression model with P-value as the screening criterion and the least absolute shrinkage and selection operator (LASSO), both of which belong to regression-based screening methods and have been widely used [44].

1. A stepwise logistic regression model with P as the screening criterion: The principle is: Firstly, all models are introduced to construct a regression model, and then all independent variables are sorted using the screening criteria, and the independent variable with the least correlation of the dependent variable is eliminated from the model[45]. The P-value is usually set to include and exclude two thresholds as variable filtering criteria. In this study, we conducted a multivariate logistic regression analysis with stepwise method (${{\varvec{\upalpha}}}_{\mathbf{i}\mathbf{n}}=0.05,{{\varvec{\upalpha}}}_{\mathbf{o}\mathbf{u}\mathbf{t}}=0.1$) to select variables, with the presence of diabetes mellitus as the dependent variable.
2. LASSO[46] is also a model based on linear regression: The principle is to regularize the coefficients by imposing an L1 penalty term on the regression coefficients, and the sum of the absolute values of the regression coefficients is less than 1 after the L1 penalty. According to this property of the L1 regularization, some regression coefficients will be punished as 0, and then they will be removed from the model, so the regression model variable screening function is also given.

#### Random forest

Random Forest (RF) is an ensemble method, which is based on decision tree [47]. RF reduce the degree of overfitting by combining multiple overfit evaluators (ie, decision trees) to form an ensemble learning algorithm. Each decision tree can get the corresponding classification decision result. By using the voting results of each decision tree in the forest, the category of the sample to be tested is determined according to the principle of minority obeying the majority, and the category with higher votes in all decision trees was determined to be the final result.

#### Gradient boosting (GBDT)

Friedman [48] proposed the Gradient Boosting model in 1999. Its basic idea is to first initialize the model and determine the loss function, calculating the pseudo residual under the original model, creating a learner to explain the pseudo residual, reducing the pseudo residual in the gradient direction, multiplying the learner by the weight coefficient and linearly combining with the original model to form a new model and iterating repeatedly. The key is that each new iteration is to reduce the residual of the previous iteration, so that the model could proceeds along the direction of the fastest residual reduction, resulting in a series of weak classifiers, each of which is a binary tree. These weak classifiers would be combined to form a model that could minimize the loss function.

#### Support vector machine (SVM)

Support Vector Machine (SVM) [49] is based on the theory of VC dimension and the theory of minimum structural risk in statistical theory. It maps data points to a high-dimensional space (Hilbert space) through a kernel function, making linearly inseparable data linearly separable. Also, it helps establish the maximum separation and optimal separation hyperplane in the feature space to maximize the distance between the optimal hyperplane and the two types of samples. The structure risk minimization idea makes the classifier experience risk and generalization error smaller.

#### Logistic regression

Logistic Regression (LR) algorithm [50], mainly used in two classification problems. LR algorithm is widely used in disease diagnosis because of its fast calculation speed, good interpretability, easy expansion and easy implementation. The LR algorithm uses the Sigmoid function as the prediction function. The input variable x outputs the variable y through the linear function$\mathrm{y}=\mathrm{ax}+\mathrm{b}$, and then the output variable y is converted into the labeled result through the Sigmoid function. The threshold of the model function, an adjustable parameter, would first be set, and the model will judge the sample value as category "1" when the output value of the Sigmoid function is greater than the threshold, otherwise, it will be judged as category "0". To prevent the model from overfitting, the LR algorithm will add a regularization term to the cost function of logistic regression to obtain a more suitable machine learning model. Common regularization methods consist of L1 regularization and L2 regularization. “C” (the reciprocal of the regularization coefficient) is another important parameter affecting the performance of the LR algorithm.

#### Evaluation index

Considering the advantage of accuracy is that it can intuitively explain the possibility of correctly predicted samples, but in the real world, the distribution of sample data often has the problem of unbalanced categories. When there is a category imbalance, using accuracy as a measurement criterion will lead to a situation where the accuracy of the model is high but the prediction ability is insufficient. For this reason, this study uses multiple indicators such as accuracy, precision, recall, and F1 score to jointly evaluate the performance of the prediction classification of the four classifiers of GBDT, random forest, Logistic Regression and SVM. The significance of each evaluation index is shown in Table S3 (Additional file 1: Table S3).

Confusion matrix: Each column of the matrix represents the predicted classification of the sample, while each row represents the true classification of the sample. In the end, each cell is a possible combination of predicted classification and true classification. See Table S4 (Additional file 1: Table S4) for details.

#### Statistical analysis

IBM SPSS Version 22 (IBM Corp., Armonk, NY, USA) was used for statistical description and logistic regression analysis of the data. Significance for all statistical tests was a priori at P < 0.05 and all P values were two-tailed; Python (version 3.7.2) was used for LASSO variable screening, SVMSMOTE resampling, and the construction and optimization of each classifier model.

## Results

### Experimental setup

To determine whether the feature selection methods and SVM-SMOTE resampling method improve classification, several phases need to be completed. Firstly, we classify the initial full dataset (unbalanced data set) with all the features. Secondly, use each of the two variable selection methods (Logistic stepwise regression and Lasso feature screening) for feature selection, and obtain a new reduced data set for each method. These new data sets will introduce four classifiers: Random Forest, GBDT, Logistic Regression, and SVM to generate new prediction results. Thirdly, based on feature screening, combined with SVM-SMOTE resampling technology, the data set after feature screening is equalized, and then the above four classifiers are introduced again to generate new prediction results. In these steps, we have been able to observe whether these methods themselves increase or decrease efficiency. In terms of model verification, we randomly selected 70% of the data as training data and the remaining 30% as test data. To ensure the stability of the model, we recycle the data segmentation and model setting process 100 times and use the average of 100 test results as the final predicted value of the evaluation model. Accuracy, precision, recall, F1-Score, and AUC were used to evaluate the performance of each model. Finally, the feature selection method, resampling method and classifier algorithm that make the model performance the best are selected to construct the combined model.

### Feature selection

Given the redundant information that might make the classification results of diabetes unsatisfied in chronic disease survey data, the feature dimension reduction methods, namely Logistic stepwise regression and LASSO, were adopted to retain relevant information and deduct irrelevant information. Logistic stepwise regression was carried out in SSPS 22.0, finally, 6 variables enter the model, such as age (OR 1.194, 95% CI 1.005–1.419), region (OR 1.647, 95% CI 1.327–2.045), heart rate (OR 1.462, 95% CI 1.128–1.895), BMI (OR 1.384, 95% CI 1.198–1.599) whether it is diagnosed as hypertension (OR 1.901, 95% CI 1.507–2.398), hyperlipidemia (OR 1.318, 95% CI 1.059–1.639). According to the OR value, it can be known that hypertension, heart rate, and region are high-risk factors that affect blood glucose elevation (Table 2). The risk of diabetes in patients with hypertension is 1.901 times that of those with normal blood pressure; those with abnormal heart rate are 1.462 times of those with normal heart rate; the risk of diabetes among urban residents is 1.647 times that of rural residents.

**Table 2:** Logistic regression analysis results

Factors	$$\overset{\lower0.5em\hbox{$\smash{\scriptscriptstyle\frown}$}}{\beta }$$	SE	Wald $${}^{2}$$	P	OR	OR(95%CI)	
						Lower	Upper
Age(${\mathrm{x}}_{2}$)	0.177	0.088	4.073	0.044	1.194	1.005	1.419
Region(${\mathrm{x}}_{3}$)	0.499	0.110	20.494	< 0.001	1.647	1.327	2.405
Heart rate(${\mathrm{x}}_{14}$)	0.308	0.132	8.256	0.004	1.462	1.128	1.895
BMI(${\mathrm{x}}_{15}$)	0.325	0.074	19.528	< 0.001	1.384	1.198	1.599
Hypertension (${\mathrm{x}}_{17}$)	0.642	0.119	29.384	< 0.001	1.901	1.507	2.398
Hyperlipidemia(${\mathrm{x}}_{18}$)	0.276	0.111	6.138	0.013	1.318	1.059	1.639
Constant	− 3.783	0.195	376.027	< 0.001	0.023		

**Fig. 1:**
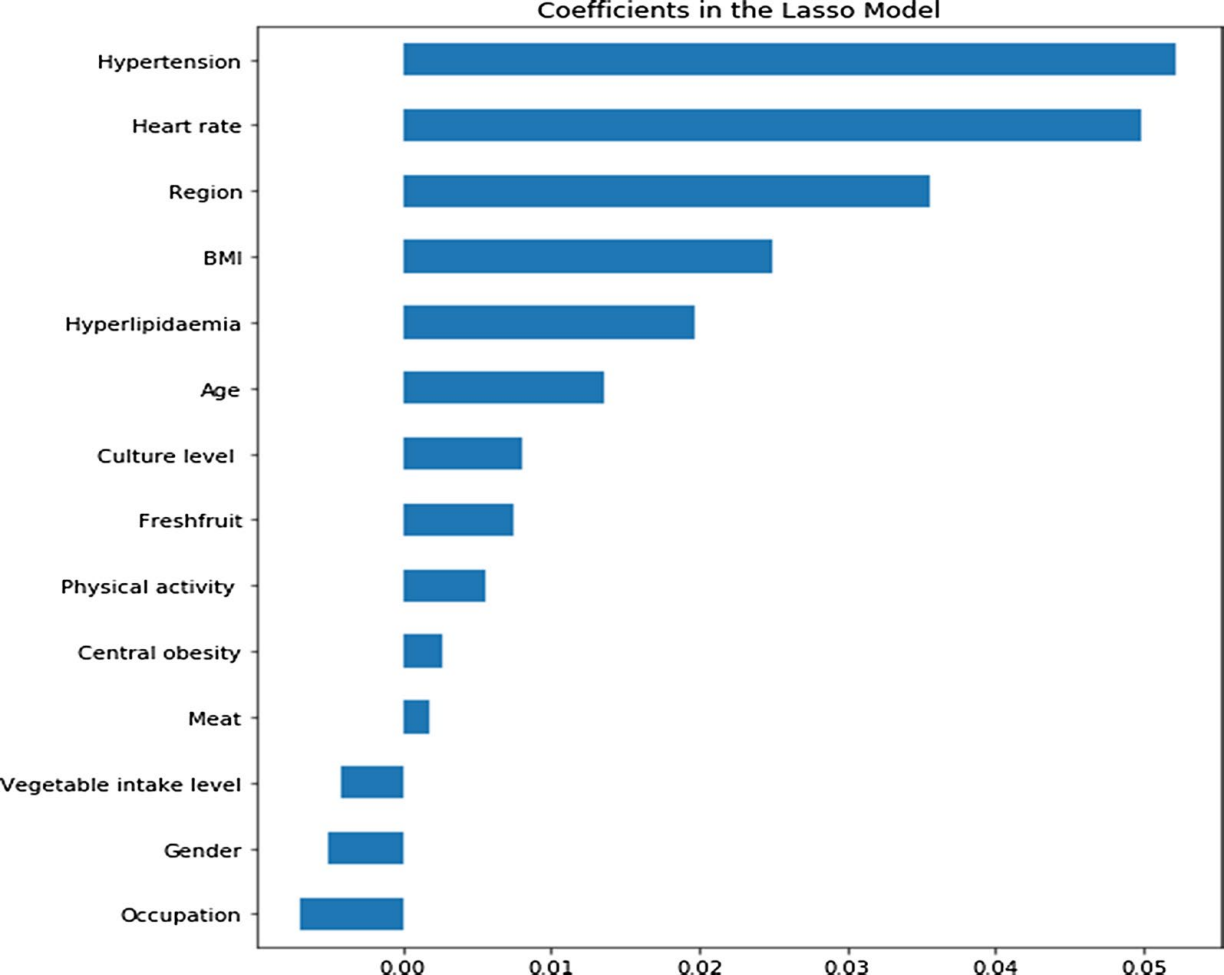
Variable importance ranking based on LASSO feature selection

LASSO will be implemented by “LassoCV” statements in Python software. For the key parameter λ of regularization, a ten-fold cross-validation method is used to select the value that maximizes the performance of the model (λ = 0.001), and finally the feature coefficient with the lowest impact is reduced to 0 and eliminated, and the final 14 variables are obtained (Fig. 1a) Shown).

### Correlation analysis between variables

**Fig. 2:**
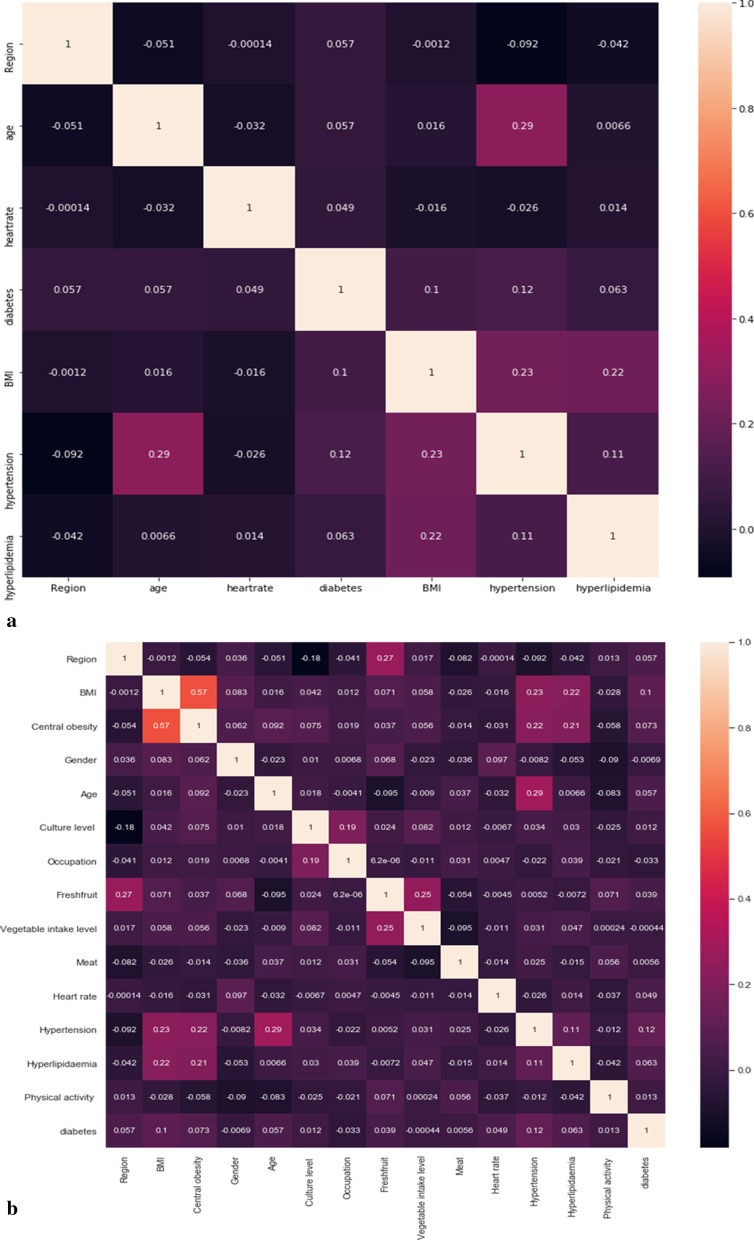
Heat map of the correlation coefficient of variables that finally enters the model after dimensionality reduction based on Logistic stepwise regression (**a**) and LASSO (**b**) features

In order to prevent the collinearity between the variables from affecting the performance of the model, this study performed the correlation analysis between the variables on the feature sets selected by the two feature screening methods. The correlation coefficient heat map between factors was drawn based on the correlation coefficient, and Fig. 2a, b was obtained. It can be seen from the Fig. 2 that the correlation coefficients among the 6 variables selected by Logistic Regression are relatively small (all ≤ 0.29); among the 14 variables screened by LASSO, only the correlation between BMI and central obesity is relatively large, with a correlation coefficient of 0.57, and the correlation coefficients among the remaining variables are all lower than 0.3. Therefore, according to the importance of factors to hyperglycemia (See Fig. 1 for details), the BMI is retained and the variable of central obesity is eliminated. In the end, 13 variables including age, region, heart rate, hypertension, hyperlipidemia, culture level, fresh fruit, physical activity, meat, vegetable intake level, gender, and occupation were selected by the LASSO method to enter the model.

In the ranking of the importance of variables obtained by the LASSO method, the first six variables are almost the same as the ranking of the importance of the six final variables selected in the Logistic regression (See Table 2 and Fig. 1 for details).

### Model establishment and evaluation

#### Parameter optimization and selection of important parameters

For the hyperparameters of each classification algorithm, we first execute Grid search with tenfold CV within the training set to determine the optimal hyperparameters. Then, we use the entire training set to train the model with the optimal hyperparameters and assess the trained model in the testing set. For Logistic Regression classification, we tune the parameters based on penalty and “C”; for SVM classification, we tune the parameters based on kernel, gamma and “C”; for GBDT classification, we tune the parameters based on n_estimators and learning_rate; for Random Forest classification, we tune the parameters based on n_estimators, max_depth and max_features. The adjusted parameters and final values are shown in Table 3.

**Table 3:** Parameter selection and optimization

Classification algorithm	Parameters	Parameter selection range	Final value
LR*	L	L1, L2	L2
	C	1 to 10, step size 0.01	3.95
SVM	Gamma	0 to 1, step size 0.01	0.06
	C	1 to 10, step size 1	1.00
	Kernel	linear, rbf, sigmoid	rbf
GBDT	Learning-rate	0 to 1, step size 0.01	0.06
	n_estimators	10 to500, step size 10	60
RF*	n_estimators	10 to 500, step size 10	130
	max_depth	1 to15, step size 1	2
	max_features	1 to 15, step size 1	2

*LR = Logistic Regression; RF = Random Forest;

### Model performance evaluation

With the dataset above, we randomly sampled 70% of the data as training data and the remaining 30% as test data. To reduce the variability caused by the data partition, we recycle the data segmentation and model setting process 100 times, and use the average of 100 test results as the final predicted value of the evaluation model. The results are presented in Table 4. It can be seen from Table 4 that on the complete feature set (using 18 attribute features as input variables, and whether the patient has diabetes or not as output variables), each classification model showed a phenomenon that the overall accuracy of the model was high (all above 90%), while the recognition rate of positive samples was extremely low. The reason may be the poor performance of the classifier due to collinear correlation features and extremely unbalanced data. Next, we will discuss in detail the impact of feature selection and class balance processing methods on classification model performance.

**Table 4:** Average and dispersion of 100 times hold-out test results

	AUC		Accuracy		Precision		Recall		F1-Score	
	Mean	Std	Mean	Std	Mean	Std	Mean	Std	Mean	Std
LR	0.649	0.020	0.907	0.007	0.085	0.277	0.001	0.003	0.002	0.005
Logistic-LR	0.657	0.019	0.908	0.007	0.02	0.141	0.0002	0.001	0.0003	0.002
LASSO-LR	0.654	0.020	0.907	0.007	0.15	0.360	0.001	0.003	0.003	0.006
Logistic-SVMSMOTE-LR	0.675	0.009	0.630	0.108	0.622	0.013	0.663	0.028	0.642	0.014
LASSO-SVMSMOTE-LR	0.657	0.009	0.621	0.009	0.616	0.013	0.639	0.016	0.627	0.010
SVM	0.555	0.025	0.907	0.007	0	0	0	0	0	0
Logistic-SVM	0.500	0.034	0.907	0.007	0	0	0	0	0	0
LASSO-SVM	0.546	0.023	0.907	0.007	0	0	0	0	0	0
Logistic-SVMSMOTE-SVM	0.715	0.009	0.660	0.011	0.630	0.014	0.777	0.026	0.696	0.012
LASSO-SVMSMOTE-SVM	0.839	0.008	0.763	0.009	0.737	0.012	0.816	0.014	0.775	0.009
GBDT	0.632	0.022	0.903	0.008	0.133	0.167	0.006	0.007	0.012	0.012
Logistic-GBDT	0.630	0.019	0.904	0.008	0.115	0.187	0.004	0.006	0.008	0.012
LASSO-GBDT	0.629	0.019	0.903	0.007	0.128	0.156	0.007	0.018	0.013	0.010
Logistic-SVMSMOTE-GBDT	0.750	0.009	0.691	0.010	0.673	0.013	0.743	0.018	0.706	0.010
LASSO-SVMSMOTE-GBDT	0.841	0.007	0.765	0.008	0.743	0.011	0.811	0.013	0.775	0.008
RF	0.600	0.021	0.906	0.007	0.180	0.295	0.003	0.004	0.006	0.009
Logistic-RF	0.559	0.018	0.902	0.008	0.244	0.154	0.020	0.011	0.037	0.019
Lasso-RF	0.602	0.019	0.901	0.008	0.151	0.094	0.0128	0.008	0.023	0.015
Logistic-SVMSMOTE-RF	0.811	0.008	0.743	0.008	0.706	0.012	0.834	0.020	0.764	0.008
LASSO-SVMSMOTE-RF	0.948	0.004	0.890	0.005	0.869	0.009	0.919	0.007	0.893	0.006

### Model performance comparison after feature dimensionality reduction only

**Fig. 3:**
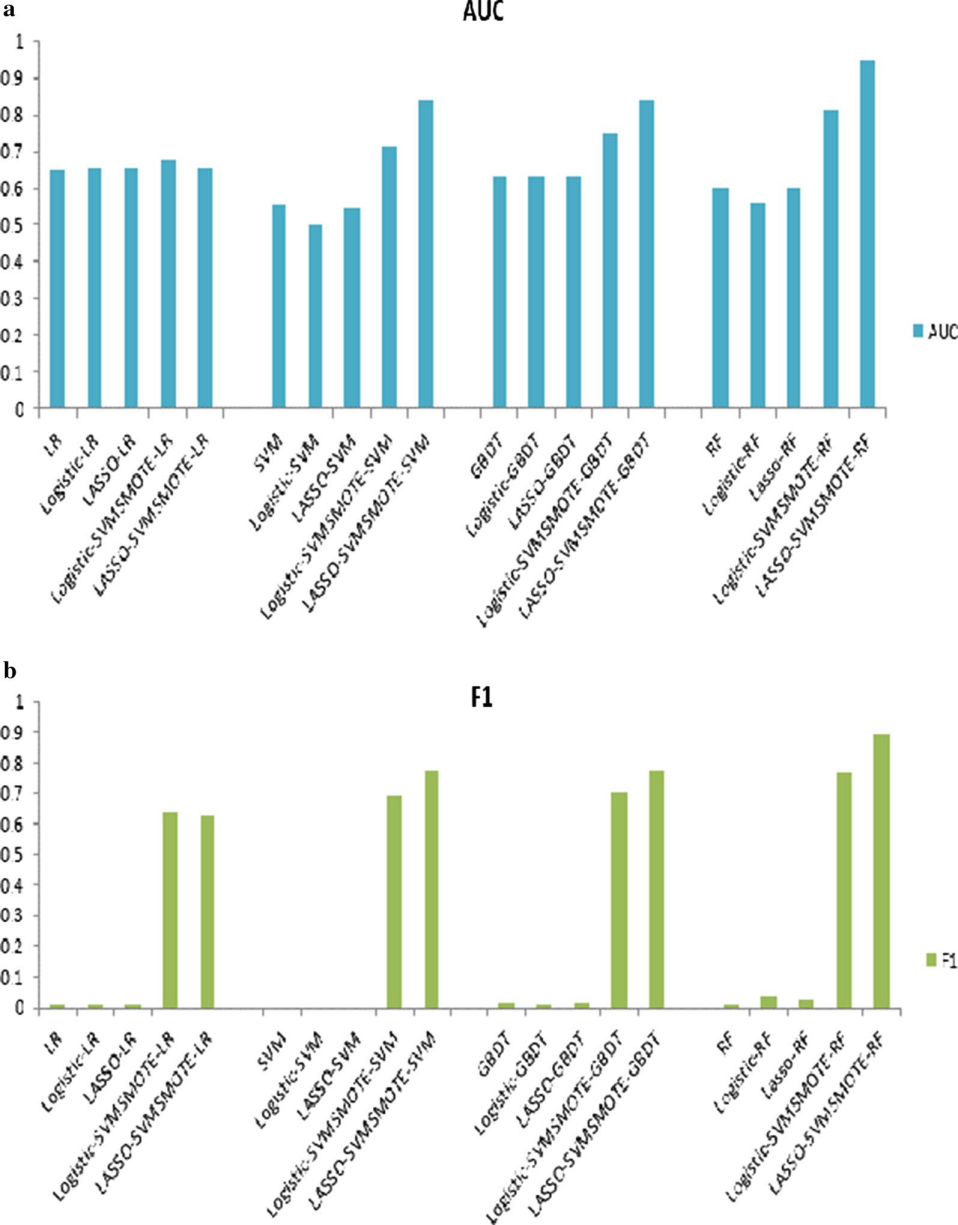
Performance analysis of each model based on F1-Score (**a**) and AUC (**b**)

According to the indices’ values, after feature screening, none of the classification models showed the best classification performance on all evaluation indicators. Since the diagnosis of diseases is more focused on finding positive cases, this article uses Recall, AUC, and F1-Score as examples to visualize the test results of each model. Combining Table 3 and Fig. 3, it could be seen that the performance of some classifiers has been improved. For example, the AUC of Logistic Regression in the two simplified feature data sets was higher than that of the full feature set, with 0.657, 0.654 and 0.649 respectively. The RF was better than before feature screening on F1-Score the complete feature set, with 0.037, 0.023 and 0.006 respectively. Although the classification performance of each classifier had not changed much on the whole, on the basis of feature selection, it not only simplifies the model complexity, but also ensures the predictive ability of each model to a certain extent, and even improves the model performance. Explain that feature selection is necessary.

### The classification performance of DM combined with resampling and dimensionality reduction

To further improve the classification performance of the model for DM, we further combined the SVM-SMOTE resampling technology on the two feature sets extracted by the two feature screening methods to evaluate the classification performance of the four classifiers for DM classification. After resampling, the unbalance ratio of the data is converted from the original 9.53:1 to 1:1. The results have shown that after combining resampling and feature screening, the overall performance of each classifier had been improved to a certain extent, especially the balanced data after SVM-SMOTE resampling, which significantly improved the ability of each classification model to identify positive samples (see Table 4 and Fig. 3), indicating that the imbalance of data has a great impact on the prediction performance of the classification model.

According to the results of each evaluation index value, it could be seen that after combining feature screening and resampling technology, the Logistic Regression classification algorithm performed the worst, and all the index values in the test set were low. The prediction performance of the ensemble classification algorithm was generally better than that of the single classification algorithm. The Random Forest ensemble algorithm performed best among the four classifiers. Compared with the multi-factor Logistic Regression feature screening method, the feature set screened by LASSO enabled the classification models to obtain better prediction performance as a whole. Among them, LASSO-SVMSMOTE-Random Forest (Accuracy = 0.890, Precision = 0.869, Recall = 0.919, F1-Score = 0.893, AUC = 0.948) had the best overall classification performance, followed by LASSO-SVMSMOTE-GBDT (Accuracy = 0.765, Precision = 0.743, Recall = 0.811, F1-Score = 0.775, AUC = 0.811). According to the standard deviation of each index value, it could be seen that the model performance was relatively stable (especially after the data sets were processed by class balance processing, the standard deviation value was stable at about 0.01). Considering the prediction results of all classification models, we finally chose the better-performing LASSO feature screening method and SVM-SMOTE resampling method to construct a combined classification model with Random Forest.

## Discussion

The onset of DM is hidden, with early symptoms not obvious. It will not only cause serious complications but also be related to the occurrence of cancer, cognitive dysfunction, tuberculosis, depression and other diseases. Moreover, the incidence of DM in China is increasing year by year [3]. High-risk groups of DM can be early identified by specific models, which help to detect high-risk groups and thus helps to improve people’s well-being.

In this chronic disease survey data-based classification task, we explored different supervised classifiers combined with SVM-SMOTE and two-dimensionality reduction methods (Logistic Stepwise Regression and LAASO) on the classification of DM with an unbalanced class of samples. The results of four supervised classifiers based on four data processing approaches were discussed. Accuracy, Precision, Recall, F1-Score and AUC values were selected as critical indicators to evaluate the performance of classification models. The results in this study showed that the Random Forest classifier combining with SVM-SMOTE and LASSO feature reduction method obtained the best performance in distinguishing people at high risk of DM from normal samples. In the classification performance comparison after the separate feature screening process, it is concluded that feature reduction can not only simplify the model complexity to a certain extent, but also improve the model performance. After the combination of SVM-SMOTE re-sampling processing, the performance of each model has been further improved. At the same time, we found that ensemble classifiers, such as Random Forest classification and GBDT classification, have a more significant performance improvement than single classifiers (Logistic Regression and SVM) in the balanced data after combined resampling processing. Random Forest classification performed best. Since the ensemble classification model allows to balance noise from diversified models and enables to strengthen the generalization ability, it has better prediction results, which have been verified in the application research of many ensemble models. Also, the combined model combined with LASSO feature dimensionality reduction is better than the combined model based on Logistic stepwise regression feature dimensionality reduction. This may be related to the logistic regression method that excludes useful information when modelling, while LASSO retains more characteristic variables and does not increase the collinearity between variables.

In view of the high-dimensional feature space and high feature redundancy of medical data, it is necessary to perform feature selection operations when mining medical data. We choose Logistic stepwise regression and LASSO, which are commonly used in previous studies, for feature dimensionality reduction. After Logistic stepwise regression screening, six variables finally entered the model (as shown in Table 3). LASSO originally retained 14 features. A heat map of the correlation coefficient between variables was further drawn to move out the negative effect on the model caused by collinearity for it retains more variables. For the high-correlated BMI and central obesity (correlation coefficient = 0.57), the BMI mostly impacting the outcome variables are retained; 13 variables entered the model at last (Figs. 1, 2b). The importance ranking of the most important first six variables is almost consistent with the results of Logistic stepwise regression. This is relatively consistent with the results of previous studies on DM-related factors [51, 52], and has certain rationality. Besides, category imbalance runs into the forefront of research in machine learning and pattern recognition. When different category samples in the training data set is very different, the performance of the classification algorithm will be significantly reduced. As shown in the research in this article, the significant difference between various methods is mainly the usage of SVM-SMOTE resampling technology. The balanced improve the performance of each classifier. It shows that the unbalanced distribution as a priori information has a strong influence on the final discrimination in many cases. The SVM-SMOTE method pays more attention to some minority samples at the boundary of the optimal decision function, which makes the newly generated sample distribution more reasonable. Hien M. Nguyen et al. in 2011 proved the superiority of this method in the experimental research on boundary oversampling method in the classification of unbalanced data [42].

Random Forest enjoys high classification accuracy, fast operation speed, and good robustness. CASANOVA et al. [53] once used Jackson heart research cohort data and found that the prediction accuracy of the Random Forest algorithm is higher than the multi-factor Logistic Regression analysis. Pradeep Kandhasamy et al. [54] used public data in the UCI machine learning data repository to verify that the prediction accuracy of the random forest algorithm is higher than that of Support Vector Machines and KNN, which is consistent with the results of this paper. Ding et al. [55]used the Random Forest algorithm for somatic mutation detection on the tumor normal paired sequence data set, and obtained a prediction accuracy of 92.28%, which is better than the 91.6% and 86.77% of the Support Vector Machine and Logistic Regression. In summary, Random Forest is an excellent integrated machine learning algorithm, and its classification ability has been verified in many research fields. However, like other classifiers such as SVM, KNN, ANN, etc., when faced with the high-dimensional feature space of medical data, there are a large number of redundant features, noise features and sample category imbalance, etc., the classification performance and execution time of the Random Forest algorithm encounter new challenges. Therefore, based on the basic idea of combining Random Forest algorithm, equilibrium processing of unbalanced data and feature selection, the research and design of Random Forest combination method which can effectively deal with the problem of high-dimensional data and category imbalance data in medical data mining is still a subject with strong innovation and high research value. The results of this experiment also fully proved that the Random Forest combined classification model combining feature dimensionality reduction and resampling technology enjoy the best classification effect.

However, several problems stand out with the establishment of further models. Firstly, the hyperparameters of the classification model in this study are optimized by grid search algorithm, and the rest are optimized by the software default parameters. In future research, we will focus on exploring parameter optimization methods to better improve the performance of the model. Secondly, there is a lack of family history in the investigation of risk factors for hyperlipidemia, and there are certain limitations in the indicators of bad living behaviors.

## Conclusions

In this paper, the application of information about lifestyle, physical condition and diet in the classification of DM was discussed through different combinations of feature dimensionality reduction methods, SVM-SMOTE, and supervised classifiers. The results showed that the Random Forest classifier combining with SVM-SMOTE and LASSO feature reduction method performs best in telling high-risk patients of DM from ordinary individuals. Besides, it is worth mentioning that we systematically presented the diagnosis performance of each classification model after implementing dimensionality reduction and/or resampling.

## Supplementary Information

**Additional file 1:** **Table S1**. Sampling process of survey subjects for chronic disease surveillance in China; **Table S2**. Survey subject general information; **Table S3**. Classification model evaluation index; **Table S4**. Confusion Matrix

**Additional file 2:** Questionnaire (in English)

## Data Availability

The data that support the findings of this study are available from the corresponding author upon reasonable request.

## Abbreviations

DM: Diabetes Mellitus
LASSO: Least absolute shrinkage and selection operator
LR: Logistic regression
RF: Random forest
GBDT: Gradient boosting
SVM: Support vector machine
SMOTE: Synthetic minority oversampling technique
OR: Odds ratio
AUC: Area under the ROC Curve
BMI: Body mass index

## References

[1] Herman WH (2017). The Global burden of diabetes: an overview.

[2] Zhang M, Zhou J, Liu Y, Sun X, Luo X, Han C, Zhang L, Wang B, Ren Y, Zhao Y (2017). Risk of type 2 diabetes mellitus associated with plasma lipid levels: The Rural Chinese Cohort Study. Diabetes Res Clin Pract.

[3] Carracher AM, Marathe PH, Close KL (2018). International Diabetes Federation 2017. J Diabetes.

[4] Gu W, Ren Y, Ji L (2016). Non-linear associations of risk factors with mild hypoglycemia among Chinese patients with type 2 diabetes. J Diabetes Complications.

[5] Guidelines for the prevention and control of type 2 diabetes in China (2017 Edition). *Chin J Pract Internal Med *2018; 38(4):292–344.

[6] Haijian G, Changping J, Zilin S, Xiaoning L, Suixia Y, Tao M, Ya S, Chen Q, Juan C, Xuepeng X (2017). An analysis of the quality of life among residents with type 2 diabetes mellitus, pre-diabetes mellitus or normal blood glucose. Chin J Diabetes.

[7] Fodor JG, Adamo KB: Prevention of Type 2 Diabetes Mellitus by Changes in Lifestyle. *New England Journal of Medicine* 2001, 345(9):696; author reply 696–697. 10.1056/NEJM200108303450912 11547727

[8] Kuritzky L: Reduction in the incidence of type 2 diabetes with lifestyle intervention or metformin. (Brief Article). *N Engl J Med* 2015, 346. 10.1056/NEJMoa012512 PMC1370926 11832527

[9] Park JY, Rha SW, Choi BG, Choi JW, Ryu SK, Kim S, Noh YK, Choi SY, Akkala RG, Li H (2015). Impact of low dose atorvastatin on development of new-onset diabetes mellitus in Asian population: Three-year clinical outcomes. Int J Cardiol.

[10] Peter WFW (2005). Metabolic syndrome as a precursor of cardiovascular disease and type 2 diabetes mellitus. Circulation.

[11] Thomas A, Henrik S, Jan J, Vestergaard S (2004). The independent effect of type 2 diabetes mellitus on ischemic heart disease, stroke, and death: a population-based study of 13,000 men and women with 20 years of follow-up. Arch Intern Med.

[12] Sakellaropoulos T, Vougas K, Narang S, Koinis F, Kotsinas A, Polyzos A, Moss TJ, Piha-Paul S, Zhou H, Kardala E (2019). A Deep Learning Framework for Predicting Response to Therapy in Cancer. Cell Reports.

[13] Holzinger A, Haibe-Kains B, Jurisica I (2019). Why imaging data alone is not enough: AI-based integration of imaging, omics, and clinical data. Eur J Nucl Med Mol Imaging.

[14] Mysona DP, Tran LKH, Tran PMH, Gehrig PA, Le LV, Ghamande S, Rungruang BJ, Java J, Mann AK, Liao J (2020). Clinical calculator predictive of chemotherapy benefit in stage 1A uterine papillary serous cancers. Gynecol Oncol.

[15] Schomberg J (2019). Identification of targetable pathways in oral cancer patients via random forest and chemical informatics. Cancer Informatics.

[16] Morpurgo R, Mussi S (2002). I-DSS: an intelligent diagnostic support system. Expert Syst.

[17] Sela RJ, Simonoff JS (2012). RE-EM trees: a data mining approach for longitudinal and clustered data. Mach Learn.

[18] Hai ND, Giang NL (2013). Anomaly detection with multinomial logistic regression and Naïve Bayesian. Lecture Notes in Electrical Engineering.

[19] Gui-Jie Z, Shuai W: Decision Tree Classification. *Jilin Normal Univ J (Natural ence Edition)* 2008.

[20] Kavzoglu T (2009). Increasing the accuracy of neural network classification using refined training data. Environ Model Softw.

[21] Wikipedia F: Naive Bayes Classifier. 2016.

[22] Saunders C, Stitson MO, Weston J, Holloway R, Bottou L, Scholkopf B, Smola A (2002). Support vector machine. Computer Science.

[23] Dietterich TG (1997). Machine-learning research. AI Mag.

[24] Omar R: Clinical Prediction Models: A Practical Approach to Development, Validation and Updating by STEYERBERG, E. W. *Biometrics* 2010, 66(2).

[25] Yang Q, Wu X (2006). 10 Challenging Problems in Data Mining Research. Int J Inf Technol Decis Mak.

[26] Brown I, Mues C (2012). An experimental comparison of classification algorithms for imbalanced credit scoring data sets. Expert Syst Appl.

[27] Mutrofin S, Venantius R, Ginardi H, Fatichah C, Kurniawardhani A (2019). A critical assessment of balanced class distribution problems: the case of predict student dropout. Test Eng Manag.

[28] Mena L, Gonzalez JA: Machine Learning for Imbalanced Datasets: Application in Medical Diagnostic. In: *Nineteenth International Florida Artificial Intelligence Research Society Conference: 2006*; 2006.

[29] Galar M (2012). A review on ensembles for the class imbalance problem: bagging-, boosting-, and hybrid-based approaches. IEEE Trans Syst Man Cybern Part C Appl Rev.

[30] Sun Z, Song Q, Zhu X, Sun H, Xu B, Zhou Y (2015). A novel ensemble method for classifying imbalanced data. Pattern Recogn.

[31] Exploring different strategies for imbalanced ADME data problem: case study on Caco-2 permeability modeling. *Molecular Diversity* 2016, 20(1):93-109 10.1007/s11030-015-9649-4 26643659

[32] Barandelaa R, Sanchezb JS, Garcia V (2003). Strategies for learning in class imbalance problems. Pattern Recogn.

[33] Tahir MA, Kittler J, Yan F (2012). Inverse random under sampling for class imbalance problem and its application to multi-label classification. Pattern Recognit.

[34] Garcia S, Herrera F (2014). Evolutionary undersampling for classification with imbalanced datasets: proposals and taxonomy. Evol Comput.

[35] Chawla NV, Bowyer KW, Hall LO, Kegelmeyer WP (2011). SMOTE: synthetic minority over-sampling technique. J Artif Intell Res.

[36] Rao H, Wu E, Fu S, Yang M, Feng B, Lin A, Fei R, Fontana R, Lok A, Wei L : The higher prevalence of truncal obesity and diabetes in American than Chinese patients with chronic hepatitis C might contribute to more rapid progression to advanced liver disease. *Aliment Pharmacol Ther* 2017(8):731–740. 10.1111/apt.14273 28833342

[37] Hu M, Wan Y, Yu L (2016). Prevalence, awareness, treatment, and control of hypertension and associated risk factors among adults in Xi'an, China: a cross-sectional study. Medicine.

[38] Huang Y, Gao L, Xie X, Tan S (2014). Epidemiology of dyslipidemia in Chinese adults: meta-analysis of prevalence, awareness, treatment, and control. Popul Health Metrics.

[39] Liu X, Li Y, Li L (2016). Prevalence, awareness, treatment, control of type 2 diabetes mellitus and risk factors in Chinese rural population: the RuralDiab study. Sci Rep.

[40] Huang X, Zhou Z, Liu J, Song W, Chen Y, Liu Y, Zhang M, Dai W, Yi Y, Zhao S (2016). Prevalence, awareness, treatment, and control of hypertension among China's Sichuan Tibetan population: a cross-sectional study. Clin Exp Hypertens.

[41] Tao S, Haifeng W, Zhigang L, Wen H, Lei Z, Pingxin L, Xiuhua G (2012). Applycation of SMOTE arithmetic for unbalanced data. Beijing Biomed Eng.

[42] Nguyen HM, Cooper EW, Kamei K (2011). Borderline over-sampling for imbalanced data classification. Int J Knowl Eng Soft Data Paradigms.

[43] Sanchez-Pinto L, Venable L, Fahrenbach J, Churpek M (2018). Comparison of variable selection methods for clinical predictive modeling. Int J Med Inf.

[44] Bedogni G (2009). Clinical prediction models-a practical approach to development, validation and updating. J R Stat Soc.

[45] Alonzo TA (2009). Clinical prediction models: a practical approach to development, validation, and updating. Am J Epidemiol.

[46] Tibshirani R (1996). Regression shrinkage and selection via the lasso. J R Stat Soc Ser B.

[47] Liu Y, Wang Y, Jian Z: New machine learning algorithm: random forest. In: Third International Conference on Information Computing & Applications: 2012.

[48] Friedman JH (2002). Stochastic gradient boosting. Comput Stat Data Anal.

[49] Sain, Stephan R: The nature of statistical learning theory. Technometrics 1997;38(4):409–409.

[50] Basili VR, Briand LC, Melo WL (1996). A validation of object-oriented design metrics as quality indicators. IEEE Trans Softw Eng.

[51] Zhou X, Ji L, Luo Y, Han X, Zhang X, Sun X, Ren Q, Qiao Q (2009). Risk factors associated with the presence of diabetes in Chinese communities in Beijing. Diabetes Res Clin Pract.

[52] Zhang M, Zhou J, Liu Y, Sun X, Luo X, Han C, Zhang L, Wang B, Ren Y, Zhao Y (2018). Risk of type 2 diabetes mellitus associated with plasma lipid levels: the rural Chinese Cohort Study. Diabetes Res Clin Pract.

[53] Casanova R, Saldana S, Simpson SL, Lacy ME, Bertoni AG (2016). Prediction of incident diabetes in the jackson heart study using high-dimensional machine learning. PLoS ONE.

[54] Kandhasamy JP, Balamurali S (2015). Performance analysis of classifier models to predict diabetes mellitus. Proc Comput Sci.

[55] Ding J, Bashashati A, Roth A, Oloumi A, Tse K, Zeng T, Haffari G, Hirst M, Marra M, Condon A (2012). Feature-based classifiers for somatic mutation detection in tumour-normal paired sequencing data. Bioinformatics (Oxford, England).

