# Supplementary material for: Exploratory study on classification of diabetes mellitus through a combined Random Forest Classifier
Source: BMC Med Inform Decis Mak. 2021 Mar 20;21:105. doi: 10.1186/s12911-021-01471-4 (PMC7980612; doi:10.1186/s12911-021-01471-4)
Supplement: Supplementary file 1 — Additional file 1: Table S1. Sampling process of survey subjects for chronic disease surveillance in China; Table S2. Survey subject general information; Table S3. Classification model evaluation index; Table S4. Confusion Matrix [file 12911_2021_1471_MOESM1_ESM.docx]

| Sampling stage | Sample allocation | Sampling method |
| --- | --- | --- |
| The first stage | Randomly select eight representative monitoring points in Shanxi Province | Probability proportional to size  (PPS) |
| The second stage | Randomly select 4 townships or streets | Probability proportional to size  (PPS) |
| The third stage | Randomly select three villages/committees/organizations | Probability proportional to size  (PPS)) |
| The fourth stage | Random selection of one group containing 50 households from each selected residential committee/village/organization. | Pure random sampling |
| The final stage | Each family randomly selects 1 resident aged 18 and above who has lived in the area for more than 6 months | Kish table |

* The replacement rate of surveyed households at each monitoring point should be below 10%

Supplementary Table S1 Sampling process of survey subjects for chronic disease surveillance in China

| Factors | groups | cases | Composition ratio（%） |
| --- | --- | --- | --- |
| Region | Village | 2581 | 62.9 |
|  | City | 1524 | 37.1 |
| Gender | Male | 1748 | 42.6 |
|  | Female | 2357 | 57.4 |
| Age | <40 | 748 | 18.2 |
|  | 40~ | 2301 | 56.1 |
|  | 60~ | 1056 | 10.7 |
| Ethnic | Ethnic Han | 4095 | 99.8 |
|  | National minority | 10 | 0.2 |
| Culture level | College degree and above | 1686 | 41.1 |
|  | Junior and senior high school | 2098 | 51.1 |
|  | College degree and above | 321 | 7.8 |
| Marital status | Spinsterhood | 182 | 4.4 |
|  | Married or cohabiting | 3501 | 85.3 |
|  | Divorced, widowed or separated | 442 | 10.8 |
| Occupation | Farmer | 1976 | 48.1 |
|  | Retirees or unemployers | 297 | 7.2 |
|  | Employers | 640 | 15.6 |
|  | Other | 1192 | 29.0 |
| Smoking | NO | 3241 | 79.0 |
|  | YES | 800 | 21.0 |
| Drinking | Never drink | 3230 | 78.7 |
|  | Used to drink | 241 | 5.9 |
|  | Drink now | 634 | 15.4 |
| Physical activity | Insufficient | 1027 | 25.0 |
|  | Normal | 2052 | 50 |
|  | Sufficient | 1026 | 25.0 |
| Vegetable intake level | <400g/d | 2578 | 62.8 |
|  | 400~500g/d | 864 | 21.1 |
|  | >500g/d | 663 | 16.2 |
| Fresh fruit | <100g/d | 2457 | 59.9 |
|  | 100-200g/d | 1130 | 27.5 |
|  | >200g/d | 518 | 12.6 |
| Meat | <50g/d | 3650 | 88.9 |
|  | 50-100g/d | 139 | 3.4 |
|  | >100g/d | 316 | 7.7 |
| BMI | <18.5 | 64 | 1.6 |
|  | 18.5~ | 1533 | 37.4 |
|  | 24.0~ | 1669 | 40.7 |
|  | 28.0~ | 839 | 20.4 |
| Heart rate | Bradycardia | 139 | 3.4 |
|  | Normal | 3878 | 94.5 |
|  | Tachycardia | 88 | 2.1 |
| Central obesity | NO | 1369 | 33.4 |
|  | YES | 2736 | 66.7 |
| Hypertension | NO | 2370 | 57.7 |
|  | YES | 1735 | 42.3 |
| Hyperlipidemia | NO | 2838 | 57.4 |
|  | YES | 1667 | 42.6 |

Supplementary Table S2 Survey subject general information

| Classification model evaluation index | Paraphrase | formula |
| --- | --- | --- |
| Accuracy | The ratio of the number of correctly predicted samples to the total number of samples participating in the prediction | $\frac{TP+TN}{TP+TN+FP+FN}$ |
| Precision | Represents the percentage of samples that are correctly predicted among all samples that are predicted to be positive | $\frac{TP}{TP+FP}$ |
| Recall | The proportion of "True positive" samples to all positive samples | $\frac{TP}{TP+FN}$ |
| F1-Score | It is the weighted harmonic average of recall rate and precision rate, and it is an indicator to measure the correctness of positive prediction | *2×*$\frac{precision\times Recall}{precision+Recall}$ |
| AUC(Area Under Curve) | Sensitivity and specificity can be considered comprehensively without being affected by the class distribution |  |

Supplementary Table S3 Classification model evaluation index

|  |  | Predicted Class | |
| --- | --- | --- | --- |
|  |  | Yes | No |
| Actual  Class | Yes | True positive（TP） | False Negative（FN） |
|  | No | False Positive（FP） | True Negative（TN） |

Supplementary Table S4 Confusion Matrix
