# Supplementary material for: Exploratory study on classification of diabetes mellitus through a combined Random Forest Classifier
Source: BMC Med Inform Decis Mak. 2021 Mar 20;21:105. doi: 10.1186/s12911-021-01471-4 (PMC7980612; doi:10.1186/s12911-021-01471-4)
Supplement: Supplementary file 2 — Additional file 2: Questionnaire (in English) [file 12911_2021_1471_MOESM2_ESM.docx]

**Surveillance of chronic diseases and their risk factors in China（2013）**

**Personal questionnaire**

| Name of survey respondent：__________________ Telephone：_______________________ | |
| --- | --- |
| Name of monitoring site（County / District）： | Monitoring point code： □□□□□□ |
| Township/street name： | Township/Street Code： □ |
| Name of village/residential committee： | Village/residential committee code： □ |
| Family Code： □□ | |
| Investigator's signature：_____________ | Date：□□Month□□Day□□□□Year |
| Signature of Quality Control Officer at monitoring site：_______________ | Date：□□Month□□Day□□□□Year |
| Signature of Provincial Supervisor：_________________ | Date：□□Month□□Day□□□□Year |

**Chinese Centre for Disease Control and Prevention**

**Centre for the Prevention and Control of Chronic Non-Communicable Diseases**

**June 2013**

Survey start time (24-hour system)：□□ h□□ min

| **Part I Basic Information** | | | |
| --- | --- | --- | --- |
| A1 | Date of Birth | □□Month□□Day□□□□Year | |
| A2 | Gender | 1. Male 2. Female |  |
| A3 | Ethnic | 1. Han 2. Zhuang 3. Man 4. Hui 5. Miao 6. Uygur | 1. Yi 2. Tujia 3. Mongol 4. Chosen 5. Zang   88 other |
| A4 | Education level | 1. Not receiving formal schooling 2. Did not finish primary school 3. Primary school graduation 4. Junior high school graduation | 1. High school/junior college/technical school 2. Tertiary education 3. Bachelor's degree 4. Postgraduate and above |
| A5 | Current marital status | 1. Unmarried 2. Married 3. Cohabitation | 1. Bereaved spouse 2. Divorce 3. Separation |
| A6 | Occupation | 1. Agricultural, forestry, fishery and water industry production staff 2. Operators of production and transport equipment and related personnel 3. Commercial, service industry personnel 4. Heads of state organs, party organizations, enterprises and institutions 5. Clerical and related staff 6. Professional and technical staff 7. Military 8. Other workers 9. Current students 10. Not employed 11. Housekeeping 12. Retirees | |
| A7 | What type of health insurance do you currently have? | 1. Basic medical insurance for urban workers 2. Publicly funded medical treatment 3. Urban residents' medical insurance 4. New Rural Cooperative Medical Care 5. Commercial medical insurance 6. Other 7. Did not attend   99 Unknown | |
| A8 | What is your domicile? | 1. Local counties (districts) 2. In other districts within the municipality 3. In other counties belonging to the local municipality 4. In other municipalities within the province (autonomous region, municipality directly under the Central Government) 5. In other provinces (autonomous regions and municipalities directly under the Central Government) | |

| Part Ⅱ: Smoking status | | | | | | | | |
| --- | --- | --- | --- | --- | --- | --- | --- | --- |
| Current smoking status | | | | | | | | |
| B1 | Do you smoke now?  Every day, not every day, or no smoke? | 1. Yes, every day 2. Yes, but not every day…….………🡺 3. I used to, but now I don't……………🡺 4. Never ……………………………🡺 | | | | | B3  B8  B11 | |
|  | Used to determine the current smoking status of respondents, ask questions and select only one option. | | | | | | | |
| B2 | When did you start smoking every day?  Investigator's Note: Fill in "-9" for "I don't remember" | □□ Year(s) | | | | | | |
|  | Ask this question of current smokers who smoke every day. Ask and record answers. "Daily" refers to the use of at least one tobacco product daily or almost daily for a period of one month or more. Highlight start smoking every day, not only start smoking. | | | | | | | |
| B3 | How many cigarettes do you smoke on average per day (week)?  Investigators note:  The Daily Smoker Answer Option 1,  Non-daily smokers answer option 2 | 1. □□ cigarette(s) / day 2. □□ cigarette(s) / week 3. Do not smoke cigarettes | | | | | | |
|  | The number of cigarettes the participants smoked during the survey period, and the number of cigarettes they smoked per day, if not, the average number of cigarettes they smoked per day in a typical week. | | | | | | | |
| **Quit smoking behavior** | | | | | | | | |
| B4 | Have you ever quit smoking before? (It means to think about and do something about quitting smoking seriously.) | 1. Yes, in the last 12 months 2. Yes, 12 months ago………………….🡺 3. No…….…………………………….🡺 | | | | | | B6  B6 |
| B5 | In the past 12 months, have you used nicotine replacement therapy or other western medicine to try to quit smoking? | 1. Yes 2. No | | | | | | |
| B6 | Which of the following options best matches your ideas about quitting smoking? | 1. Plan to quit smoking within a month 2. Consider quitting smoking within 12 months 3. Will quit smoking, but not within 12 months 4. Don't want to give up smoking   99 Unknown | | | | | | |
| B7 | During the past 12 months, have you been advised to quit smoking from doctors or nurses? | 1 I haven't been to the hospital ……….🡺  2 The doctor advised giving up smoking ...🡺  3 The doctor did not advise giving up smoking………. ……🡺 | | | | | | B11  B11  B11 |
| B8 | How long have you stopped smoking?  (Investigators note: includes only the respondents completely give up smoking, also in the case of occasional smoking not included. Note that you can only fill in one item.) | 1. □□ Year(s) 2. □□ Month(s) 3. □□ Week(s) 4. □□ Day(s) | | | | | | |
|  | Ask this question of current non-smokers who have smoked in the past. Ask questions and record respondents' responses using only one unit (year, month, week, or day). Fill in the numbers in □□. The possibility that participants had smoked a tobacco product on occasion (such as smoking a cigar while attending a wedding) was not counted. If the time respondents answered included a decimal (for example, one year and two months), then it could be converted to a lower level (for example, one year and two months =14 months). "I don't remember." Fill in "-9." | | | | | | | |
|  | Investigators note: if **B8<1** year（**<12** months）**.** **……………......…......……....…**🡺 or **…...…….......…......…......……...………….......…......…......……..................…**🡺 | | | | | | | B9  B11 |
| B9 | During the past 12 months, have you been advised to quit smoking from doctors or nurses? | 1 I haven't been to the hospital  2 The doctor advised giving up smoking  3 The doctor did not advise giving up smoking | | | | | | |
| B10 | In the past 12 months, have you used nicotine replacement therapy or other western medicine to try to quit smoking? | 1 Yes  2 No | | | | | | |
| **Secondhand smoke exposure** | | | | | | | | |
| B11 | On a typical week, how many days are you exposed to secondhand smoke? (Secondhand smoke refers to the smoke exhaled by the smoker and the end of the cigarette.) | 1. Every day 2. On average 4-6 days per week 3. On average 1-3 days per week 4. No   99 Don't know/remember | | | | | | |
| **Knowledge, attitude and understanding** | | | | | | | | |
| B12 | As far as you know, does smoking cause any serious diseases? | 1. Yes 2. No……...…………….………….🡺   99 Unknown………………… …….🡺 | | | | B14  B14 | | |
| B13 As far as you know, will smoking cause the following diseases? | | | | | | | | |
|  | | | Yes | No | Unknown /uncertain | | | |
| a | Stroke (stroke, cerebral thrombus) | | 1 | 2 | 99 | | | |
| b | heart attack | | 1 | 2 | 99 | | | |
| c | lung cancer | | 1 | 2 | 99 | | | |
| B14 | As far as you know, does inhaling secondhand smoke cause any serious diseases? | 1. Yes 2. No……...…………….………….🡺   99 Unknown………………………….🡺 | | | | B16  B16 | | |
| B15 As far as you know, will inhaling secondhand smoke cause the following diseases? | | | | | | | | |
|  | | | Yes | No | Unknown /uncertain | | | |
| a | Adult heart disease | | 1 | 2 | 99 | | | |
| b | Pulmonary disease in children | | 1 | 2 | 99 | | | |
| c | Adult lung cancer | | 1 | 2 | 99 | | | |
| B16 | Do you agree that low-tar cigarettes are less harmful than regular cigarettes? | 1. Yes 2. No   99 Unknown | | | | | | |

| **Part III Alcohol consumption** | | | | | | | | | | | |  |
| --- | --- | --- | --- | --- | --- | --- | --- | --- | --- | --- | --- | --- |
| C1 | Have you had any alcohol in the last 12 months? | | | | | 1. Drank, before the last 30 days 2. Drank, within 30 days 3. Never had one ………….………….🡺 | | | | | D1 |  |
| C2 | How often have you consumed alcohol in the last 12 months? | | | | | 1. Daily 2. 5-6 days/week 3. 3-4 days/week 4. 1-2 days/week 5. 1-3 days/month 6. Less than 1 day/month | | | | | |  |
| Please answer: In the last 12 months, how often did you usually drink the following types of alcohol and how much did you usually drink in a day? | | | | | | | | | | | | |
| C3 | |  | a Whether alcohol is consumed  1 Yes，2 No | b Frequency of consumption（Fill in only 1 of these fields） | | | | | | Typical day's consumption on days when alcohol was consumed in the last 12 months | | |
|  |  |  |  | b1 days/week | | | b2 days/month | | b3  days/year |  |  |  |
|  |  | a. White wine (≥42%) | □ | □ | | | □□ | | □□□ | □□.□_liang(50g)_ | | |
|  |  | b. White wine (<42%) | □ | □ | | | □□ | | □□□ | □□.□_liang(50g)_ | | |
|  |  | c. Beer (580ml/bottle, 4%) | □ | □ | | | □□ | | □□□ | □□.□bottle | | |
|  |  | d. Yellow wine (18%) | □ | □ | | | □□ | | □□□ | □□.□_liang(50g)_ | | |
|  |  | e. Rice wine (18%) | □ | □ | | | □□ | | □□□ | □□.□_liang(50g)_ | | |
|  |  | f. Wine (10%) | □ | □ | | | □□ | | □□□ | □□.□_liang(50g)_ | | |
|  |  | g. Barley wine (3 %) | □ | □ | | | □□ | | □□□ | □□.□_liang(50g)_ | | |
|  | | 1. **For men：**   In the past 12 months, how often did you drink more than 125g of high white wine, or 175g of low white wine, or 3 bottles of beer, or 5 cans of beer, or 375g of yellow wine/rice wine, or 1.5 kg of wine, or 3 kg of barley wine in one sitting? | | | | | | 1 Daily or almost daily (≥5 days/week)  2 1-4days/week3  3 1-3 days/month  4 Less than 1 day/month  5 Never | | | | |
|  | | Male respondents were asked about the number of days in the past 12 months on which they had consumed more than 5 standard units of alcohol in a single occasion. See example table for conversion of standard drinking units. | | | | | | | | | | |
|  |  | **b. For women：**  In the past 12 months, how often did you drink more than 100g of high white wine, or 150g of low white wine, or 2.5 bottles of beer, or 4 cans of beer, or 300g of yellow/rice wine, or 1 kg of 2 taels of wine, or 2.5 kg of barley wine in one sitting? | | | 1 Daily or almost daily (≥5 days/week)  2 1-4days/week3  3 1-3 days/month  4 Less than 1 day/month  5 Never | | | | | | | |
|  | | Female respondents were asked about the number of days they had consumed more than 4 standard drinking units in a single session in the past 12 months. See example table for conversion of standard drinking units. | | | | | | | | | | |

| **Part IV Diet** | | | | | | | | | |
| --- | --- | --- | --- | --- | --- | --- | --- | --- | --- |
| D1 | In the past 12 months, how many meals did you usually eat a day? | | | □times | | | | | |
|  | | | | Dining Venue | | | | | |
|  |  |  |  | a Home | | b Canteen | | | c Restaurants |
| D2 | In the past 12 months, how many days a week did you usually eat breakfast at different places? | | | □Day(s) | | □Day(s) | | | □Day(s) |
| D3 | In the past 12 months, how many days a week did you usually have lunch at different dining places? | | | □Day(s) | | □Day(s) | | | □Day(s) |
| D4 | In the past 12 months, how many days a week did you usually eat dinner at different places? | | | □Day(s) | | □Day(s) | | | □Day(s) |
| Please recall if you have eaten any of the following foods in the past 12 months and estimate the frequency and amount of each type of food you have eaten. | | | | | | | | | |
|  | | a Do you consume the following foods?  1 Yes，2 No | b Frequency of consumption (enter only 1 of these) | | | | | | Average serving size per serving |
|  |  |  | b1  Number of times/day | | b2  Number of times/week | | b3  Number of times/month | b4  Number of times/year |  |
| D5 | Pork (recorded by raw weight) | □ | □ | | □ | | □ | □□ | □□.□liang(50g) |
|  | Refers to fresh or frozen pork that has not been specially processed (e.g. cured/sauced/smoked, etc.). | | | | | | | | |
| D6 | Beef, lamb and other livestock meat (recorded on a raw weight basis) | □ | □ | | □ | | □ | □□ | □□.□liang(50g) |
|  | Means fresh or frozen meat of domestic animals other than pork, including cattle, sheep, rabbits and dogs, that has not been specially processed (e.g. cured/sauced/smoked, etc.). | | | | | | | | |
| D7 | Fresh vegetables | □ | □ | | □ | | □ | □□ | □□.□liang(50g) |
|  | Ask survey respondents about their usual consumption of fresh vegetables, excluding special days such as holidays and business trips. Vegetables refer to all types of fresh, unprocessed vegetables. Vegetables that have been pickled, soaked or sun-dried are not included. | | | | | | | | |
| D8 | Fresh fruit | □ | □ | | □ | | □ | □□ | □□.□liang(50g) |
|  | Ask survey respondents about their usual consumption of fresh fruit, excluding special days such as holidays and business trips. Fruits refer to all types of fresh, unprocessed vegetables. Processed fruit such as canned fruit and dried fruit are not counted. | | | | | | | | |
| D9 | Sugary carbonated drinks (250ml/glass) | □ | □ | | □ | | □ | □□ | □□.□_glass(es)_ |
|  | Refers to sugary carbonated beverages commonly found in the market such as bottles and cans, such as cola and fruit-flavoured carbonated beverages, but excludes other non-sugar or low-sugar carbonated beverages such as soda, beer and sugar-free soft drinks. | | | | | | | | |
| D10 | Fruit juices/fruity drinks (250ml/glass) | □ | □ | | □ | | □ | □□ | □□.□_glass(es)_ |
|  | Any fruit and vegetable flavoured beverage prepared with fruit and vegetable juices and sugar, artificial additives, artificial flavours, excluding freshly squeezed juices, which are freshly prepared. | | | | | | | | |

| **Part V Physical Activity** | | | | | | | | |
| --- | --- | --- | --- | --- | --- | --- | --- | --- |
| The following questions are about the types of physical activity you do in a typical week (including farm work, work, housework, transport-related physical activity, recreational exercise or sports, etc.). | | | | | | | | |
| Work, agriculture and domestic physical activity | | | | | | | | |
| In the case of a student, physical activity during his or her studies falls under this section; In the case of athletes, their daily training activities shall be classified as work-related physical activities; In the case of postal workers and couriers who deliver items/goods on foot or by bicycle, their walking or cycling activities are counted as work-related physical activity.  When answering the question, 'high intensity activity' refers to an activity that requires a high level of physical effort or is capable of causing a significant increase in breathing or heart rate, while 'moderate intensity activity' refers to an activity that requires a moderate level of physical effort or causes a mild increase in breathing or heart rate. | | | | | | | | |
| E1 | | During your work, farming and household activities, are there any high-intensity activities that last for more than 10 minutes? | 1. Yes 2. No………………...…🡺 | | | | E4 | |
|  |  | For certain occupational groups that perform high intensity activities for longer periods of time, such as construction workers and professional athletes, even if they do not have a significant increase in respiration or heart rate during high intensity activity, they are still counted as high intensity activity. | | | | | | |
| E2 | | During your work, farming and household activities, how many days in a week do you usually perform these high-intensity activities? | □Day(s) | | | | | |
|  |  | **"during the week" refers to the week of intense activity, not an average of a time period.**  **1-7 days is a valid response** | | | | | | |
| E3 | | During your work, farming and household activities, how long do you cumulatively engage in these high-intensity activities during the day? | \| □□h(s)□□min(s) \| \| --- \| | | | | | |
|  | | Consider what survey respondents can recall about a typical day. Only consider high intensity activities lasting 10 minutes or more. Activities of less than 10 minutes will not be counted. For the duration of agricultural physical activity, particular attention is paid to the exclusion of breaks in between. Note that it should be a cumulative time of various high intensity activities. For example, if a person usually spends about 20 minutes of high-intensity activity at work in a day and 15 minutes doing high-intensity housework when they get home, they should have a cumulative total of 35 minutes of high-intensity activity in a day.  Verify that the respondent's answers are not exaggerated (e.g. more than 4 hours). | | | | | | |
| E4 | | During your work, farming and household activities, is there any moderate intensity activity that lasts more than 10 minutes? | 1. Yes 2. No ………………...…🡺 | | | | E7 | |
|  | | If the activity causes a mild increase in respiration and heart rate, it is considered moderate intensity activity. | | | | | | |
| E5 | | During your work, farming and household activities, how many days of the week do you do the above-mentioned moderate intensity activities? | □Days | | | | | |
|  |  | **"during the week" refers to the week of intense activity, not an average of a time period.**  **1-7 days is a valid response** | | | | | | |
|  | | During your work, farming and household activities, how long do you usually spend in a day doing these moderate intensity activities? | □□h(s)□□min(s) | | | | | |
|  | | Consider what survey respondents can recall about a typical day. Only consider moderate intensity activities lasting 10 minutes or more. Activities of less than 10 minutes will not be counted. For the duration of agricultural physical activity, particular attention is paid to the exclusion of breaks in between. Note that it should be a cumulative time of various moderate intensity activities. For example, if a person usually spends about 20 minutes of moderate intensity activity at work in a day and 15 minutes doing moderate intensity housework when they get home, they should have a cumulative total of 35 minutes of moderate intensity activity in a day.  Verify that the respondent's answers are not exaggerated (e.g. more than 4 hours). | | | | | | |
| **Transportation physical activity**  The following questions do not include agricultural physical activity and work and domestic physical activity as already mentioned above. | | | | | | | | |
| Be sure to introduce the survey respondent to transport-related physical activities before you begin your questioning. For example, walking or cycling to/from work or school, going shopping, visiting friends and relatives, or working in the fields. | | | | | | | | |
| E7 | Do you walk or cycle for at least 10 minutes when you are out and about? | | | 1. Yes 2. No ………………...…🡺 | | | | E10 |
|  | It must be a continuous walk or cycle ride of at least 10 minutes.  For postal workers and couriers who deliver items/goods on foot or by bicycle, their walking or cycling activities are counted as work-related physical activity. For walking or cycling for the purpose of physical exercise, it is counted as recreational physical activity.  If the answer is "No", go to E10. | | | | | | | |
| E8 | U How many days in a typical week do you walk or cycle for at least 10 minutes when you go out? | | | □Days | | | | |
|  | **1-7 days is a valid response.** | | | | | | | |
| E9 | U How long do you walk or cycle in a typical day? | | | □□h(s)□□min(s) | | | | |
|  | Refers to the cumulative time spent on walking or cycling activity in a day on days when the survey respondent walked or cycled for at least 10 minutes.  Verify that the respondent's answers are not exaggerated (e.g. more than 4 hours). | | | | | | | |
| **Recreational physical activity**  The following questions do not include the physical activities of an agricultural nature, work, household chores and transport already mentioned above. | | | | | | | | |
| Survey respondents are introduced to recreational physical activity, including all sports and exercise that are not geared towards competition. It is important to note that this refers to activities that are carried out on a regular basis rather than occasionally, and does not include any of the activities already asked about above. | | | | | | | | |
| E10 | Do you engage in high-intensity activities that last at least 10 minutes and cause a significant increase in breathing and heart rate? For example, long-distance running, swimming, playing football, etc. | | | 1 Yes  2 No ………………...…🡺 | | | | E13 |
|  | If the activity causes a significant increase in breathing and heart rate, it is considered high intensity exercise.  If the answer is "No", then go to E13. | | | | | | | |
| E11 | U How many days in a typical week do you do the above-mentioned high-intensity sports or leisure activities? | | | □Days | | | | |
|  | **1-7 days is a valid response.** | | | | | | | |
| E12 | U In a typical day, for how long do you cumulatively engage in the above-mentioned high-intensity exercise or leisure activities? | | | □□h(s)□□min(s) | | | | |
|  | Refers to the cumulative time spent in a day doing high-intensity recreational physical activity on days when the survey respondent did high-intensity recreational physical activity lasting at least 10 minutes.  Verify that the respondent's answers are not exaggerated (e.g. more than 4 hours). | | | | | | | |
| E13 | Do you engage in moderate intensity exercise and leisure activities lasting 10 minutes or more that cause a mild increase in breathing and heart rate? For example, brisk walking, tai chi, etc. | | | 1. Yes 2. No ………………...…🡺 | | | | E16 |
|  | If the activity causes a mild increase in respiration and heart rate, it is considered moderate intensity activity. | | | | | | | |
| E14 | U How many days in a typical week do you do the above-mentioned moderate-intensity exercise or leisure activities? | | | □Days | | | | |
|  | **1-7 days is a valid response.** | | | | | | | |
| E15 | U In a typical day, for how long do you cumulatively engage in the above-mentioned moderate-intensity exercise or leisure activities? | | | □□h(s)□□min(s) | | | | |
|  | Refers to the cumulative time spent in a day doing moderate-intensity recreational physical activity on days when survey respondents engage in moderate-intensity recreational physical activity that lasts at least 10 minutes.  Verify that the respondent's answers are not exaggerated (e.g. more than 4 hours). | | | | | | | |
| **Total static behaviour** | | | | | | | | |
| E16 | In a typical day, how much time do you spend sitting, leaning or lying down? (includes time spent sitting, working, studying, reading, watching TV, using the computer, resting, and all other static behaviours, but not sleeping time) | | | | | □□h(s)□□min(s) | | |
|  | Consider the total time spent sitting at work, reading, watching TV, using the computer, resting, etc., but not including time spent sleeping. | | | | | | | |
| **Static behaviour in spare time** | | | | | | | | |
| E17a | In your spare time, how much time do you spend sitting or lying down watching TV per day? | | | | | □□h(s)□□min(s) | | |
|  | The cumulative time spent sitting, leaning or lying down watching TV does not include time spent watching TV while doing something or exercising at the same time. | | | | | | | |
| E17b | In your spare time, how much time do you spend sitting or lying down using computers (including desktops, laptops, tablets, etc.) per day | | | | | □□h(s)□□min(s) | | |
|  | Including spare time to use the computer to surf the Internet, play games, watch movies, office and other static computer use behavior, excluding standing still. Computers include desktop, laptop, tablet, ebook, MP4, etc. | | | | | | | |
| E17c | In your spare time, how much time do you spend sitting or lying down using your mobile phone every day ? | | | | | □□h(s)□□min(s) | | |
|  | This includes static mobile phone use such as using a mobile phone to make calls, surf the internet, play games, read, etc. in your spare time. Standing still is not included. | | | | | | | |
| E17d | In your spare time, how much time do you spend sitting, leaning or lying down to read (paper books) on average each day? | | | | □□h(s)□□min(s) | | | |
|  | This refers only to the static act of reading paper books, newspapers and materials. Standing still is not included. | | | | | | | |
| **Sleep behaviour** | | | | | | | | |
| E18 | How many hours do you sleep in a typical day? | | | | | □□h(s)□□min(s) | | |
|  | Fill in the cumulative length of sleep in a typical day, including naps. | | | | | | | |

| **Part Ⅵ Weight, blood pressure, blood sugar, blood lipids and other information** | | | | | | | | | |
| --- | --- | --- | --- | --- | --- | --- | --- | --- | --- |
| **F1** Weight and Control | | | | | | | | | |
| F1a | When was the last time you had your weight measured? | | | | | 1. Never measured 2. Within 7 days 3. Within 1 month 4. Within 3 months 5. Within 6 months 6. Within 12 months 7. Within 12 months ago   99 I can't remember | | | |
| F1b | How has your weight changed from 12 months ago? | | | | | 1. An increase of 2.5 kg or more 2. Basically stay the same (increase or decrease within 2.5 kg) 3. Drop by more than 2.5 kg   99 Unknown | | | |
| F1c | What do you think of your current weight status? | | | | | 1. Underweight 2. Normally 3. Overweight 4. Obese | | | |
| F1d | Have you taken steps to control your weight in the past 12 months? | | | | 1. Measures were taken to lose weight 2. Measures were taken to maintain weight 3. Measures were taken to gain weight…….🡺 4. Without taking any measures to………….…🡺 | | | | F2a  F2a |
|  | Measures are a series of planned, proactive behaviours aimed at weight control and should not last less than 1 week. | | | | | | | | |
| F1e | What are some of the ways you control or reduce your weight? (multiple choices） | | | | 1. Control diet 2. Exercise 3. Drugs   88 Others | | | | |
|  | Control diet: reduce the intake of carbohydrates and fats, increase the intake of fruits and vegetables, adjust the dietary structure, etc.  Exercise: mainly refers to regular physical exercise, occasionally a cannot be counted;  Drugs: taking medicine as prescribed by a doctor or buying drugs to control weight;  All measures should be with weight control as the ultimate goal, if the ancillary activities of other purposes, cannot be counted as weight control methods. | | | | | | | | |
| **F2** Blood pressure and its control | | | | | | | | | |
| F2a | When was the last time your blood pressure was measured? | | | | 1. 7 days 2. Within 1 month 3. Within 6 months 4. Within 12 months 5. Within 12 months ago 6. Never had a blood pressure test……. 🡺   99 Don’t remember | | | | F3a |
|  | This includes measuring your blood pressure with any type of sphygmomanometer, either by your doctor or by yourself. | | | | | | | | |
| F2b | Do you know your blood pressure? | | | | 1. Above the normal range 2. Normal range 3. Below the normal range   99 Unknown | | | | |
|  | The participants' knowledge of their own blood pressure. | | | | | | | | |
| F2c | Have you ever been diagnosed with high blood pressure? | | | | 1. Yes 2. No…………………….🡺 | | | | F3a |
|  | Diagnosis made by a doctor at a health care facility. | | | | | | | | |
| F2d | The highest level of medical care where you have been diagnosed with hypertension is: | | | | 1. Provincial and above hospitals 2. Prefecture-level (city) hospitals 3. County (district) hospitals 4. Township health centers (community health service centers) 5. Village clinics (community health service stations, private clinics)   99 Unknown | | | | |
|  | On a single choice, fill in the highest rank of the health care unit that diagnosed hypertension for the respondents. | | | | | | | | |
| F2e | What measures have you taken to control your blood pressure? (multiple choices) | | | | 1. Don't take any action 2. Take medicine as directed 3. Take medicine when symptomatic 4. Control diet 5. Exercise 6. Blood pressure monitoring   88 Others | | | | |
| F2f | Have you taken any blood pressure medication in the last 2 weeks? | | | | 1. Yes 2. No | | | | |
| F2g | Have you participated in the follow-up management of hypertension provided by primary health care institutions?  (It means the community health service center, the villages and towns who gave birth to the court, village clinics regularly or not regularly check, treatment, such as dietary and exercise guidance) | | | | 1. Yes 2. No.……………………….🡺F3a   99 Unknown……………….……. 🡺 F3a | | | | |
|  | To understand the follow-up management of hypertension patients in primary medical and health institutions from the perspective of patients. Including the grassroots medical and health institutions to take the initiative to provide patients with management (such as telephone, home visit, face-to-face visit, etc.) and patients to the grassroots medical and health institutions to consult, doctors to provide guidance for them. Answer no or don't know, jump to F3a. | | | | | | | | |
| F2h | In the past 12 months, have you received any of the following examinations or instructions from a primary health care provider?  （multiple choices） | | | | 1 Measured blood pressure, □□□time(s)/year  2 Medication guidance, □□time(s)/year  3 Dietary guidance  4 Physical activity guidance  5 Quit smoking or to smoke less  6 Quit drinking or to drink less  None of these tests or guidance | | | | |
| **F3** Blood glucose and its control | | | | | | | | | |
| F3a | How long has it been since you last measured your blood sugar? | | | | 1. Within 6 months 2. Within 12 months 3. 12 months ago 4. Never had a blood sugar test…………🡺   99 Don’t remember | | F4a | | |
|  | This includes self-testing at home and going to a medical institution for testing. | | | | | | | | |
| F3b | Do you know your blood sugar? | | | | 1. Above the normal range 2. The normal range 3. Below the normal range   99 Unknown | | | | |
|  | The normal fasting or postprandial blood sugar level. Knowing one of any value is known. | | | | | | | | |
| F3c | Have you ever been diagnosed with diabetes by a doctor?  Investigators note: Gestational diabetes was not included. | | | | 1. Yes 2. No……………...………...🡺 | | F4a | | |
|  | It should be diagnosed by a doctor at a medical institution. If a female respondent said she had diabetes, she should be asked if she had it during pregnancy. If they are, they are not considered to have diabetes. | | | | | | | | |
| F3d | The highest level of care at which you have been diagnosed with diabetes is: | | 1. Provincial and above hospitals 2. Prefecture-level (city) hospitals 3. County (district) hospitals 4. Township health centers (community health service centers) 5. Village clinics (community health service stations, private clinics)   99 Unknown | | | | | | |
|  | On a single choice, fill in the highest rank of the health care unit in which the respondent was diagnosed with diabetes. | | | | | | | | |
| F3e | What measures have you taken to control your blood sugar? (multiple choices) | | 1. No measures were taken 2. Oral medication 3. Insulin injection 4. Control diet 5. Exercise 6. Blood glucose monitoring   88 Others | | | | | | |
| F3f | Have you participated in the diabetes follow-up management provided by the primary health care institution?  (It means the community health service center, the villages and towns who gave birth to the court, village clinics regularly or not regularly check, treatment, such as dietary and exercise guidance) | | 1 Yes  2 No ………………………….🡺  99 Unknown…….………….……. …🡺  | | | | F4a  F4a | | |
|  | To understand the follow-up management of diabetic patients in primary medical and health institutions from the perspective of patients. Including the basic medical and health service institutions to take the initiative to provide patients with management (such as telephone, home visit, face to face visit, etc.) and patients to the basic medical and health service institutions to consult, seek medical advice, etc. Doctors to provide guidance. Answer no or don't know, jump to F4a。 | | | | | | | | |
| F3g | In the past 12 months, grassroots health institutions doctor ever offer you the following inspection or guidance？(multiple choices possible) | | 1. Blood pressure measurement □□□times/year 2. Measuring blood glucose □□□times/year 3. Medication guidance □□□times/year 4. Dietary Guidelines 5. Physical activity instruction 6. Quit smoking or smoke less 7. Stop drinking or drink less alcohol 8. None of the above checks or guidance | | | | | | |
| **F4** Blood lipid and its control | | | | | | | | | |
| F4a | How long has it been since you last had your blood lipid measured? | | 1. Within 6 months 2. Within 12 months 3. 12 months ago 4. Never had a blood lipid test………🡺   99 Don’t remember | | | | F5a | | |
| F4b | Have you ever been diagnosed with dyslipidemia or hyperlipidemia by a doctor in a township health center or community health service center or a medical institution of higher level? | | 1. Yes 2. No.…………….……....……🡺 | | | | F5a | | |
|  | It must be diagnosed by a doctor in a township health centre or community health service centre or a hospital of higher level. | | | | | | | | |
| F4c | What measures have you taken to control your blood lipids? (multiple choices) | 1. No measures were taken 2. Take medicine as directed by the doctor 3. Control diet, 4. Exercise 5. Blood lipid monitoring as prescribed   88 Others | | | | | | | |
| **F5 Cardiovascular and cerebrovascular events** | | | | | | | | | |
| F5a | Have you ever been diagnosed with myocardial infarction by a doctor of a medical institution at or above the county / district level? | 1. Yes 2. No…...…………….……....……🡺 | | | | | | F5c | |
| F5b | When was your first diagnosis of myocardial infarction?  What month and year  or how old were you when | □□Month□□Day□□□□Year  or □□year old | | | | | | | |
| F5c | Have you ever been diagnosed as a stroke by a doctor of a medical institution at or above the county / district level? | 1. Yes 2. No…...…………….……....……🡺 | | | | | | F6a | |
| F5d | When was your first diagnosis of stroke? What month and year  or how old were you when | □□Month□□Day□□□□Year  or □□year old | | | | | | | |
| **F6 Other chronic diseases** | | | | | | | | | |
| F6a | Have you ever been diagnosed as chronic obstructive pulmonary disease (such as chronic bronchitis, emphysema) by county / district level or above medical institutions? | 1. Yes 2. No | | | | | | | |
| F6b | Have you ever been diagnosed with asthma by medical institutions at county / district level or above? | 1. Yes 2. No | | | | | | | |
| F6c | Have you ever been diagnosed as malignant tumor (including systemic malignant tumor and brain benign tumor) by medical institutions at county / district level or above? If so, where is the tumor? | 1 Not diagnosed  2 Lung cancer  3 Gastric cancer  4 Esophageal cancer  5 Liver cancer  6 Colorectal cancer  7 Breast cancer  8 Cervical cancer  9 Other | | | | | | | |
| **F7** Respiratory system status (only for subjects aged 40 and above) | | | | | | | | | |
| F7a1 | In the past 12 months, did you often cough after waking up in the morning? | 1. Yes 2. No | | | | | | | |
|  | It refers to the general situation. For example, the respondents answered "no" when they only had a cold occasionally. If they answered "yes" when they only had a cold in winter and they often had it. | | | | | | | | |
| F7a2 | In the past 12 months, did you often cough during the day or at night? | 1. Yes 2. No | | | | | | | |
|  | It refers to the cough condition at other times except in the morning under normal circumstances. For example, the respondents answered "no" only when they have a cold occasionally. If they answered "yes" only in winter but often. | | | | | | | | |
| If either of the above questions F7a1 and F7a2 is answered "yes", continue. If both of them are answered "no", skip to F7b1 | | | | | | | | | |
| F7a3 | Do you cough for three months or more every year? | 1. Yes 2. No | |  | | | | | |
|  | Only three consecutive months or more are included. | | | | | | | | |
| F7b1 | In the past 12 months, did you wake up in the morning with expectoration? | 1. Yes 2. No | |  | | | | | |
|  | Expectoration refers to phlegm in cough, excluding the clear throat. | | | | | | | | |
| F7b2 | In the past 12 months, did you often cough phlegm during the day or at night? | 1. Yes 2. No | |  | | | | | |
| If the answer to any of the above questions F7b1 and F7b2 is "yes", continue. If the answer to both questions is "no", skip to F7c | | | | | | | | | |
| F7b3 | Do you expectorate like this for three months or more every year? | 1. Yes 2. No | |  | | | | | |
| F7c | Have you ever had a lung function test? | 1. Yes 2. No   99 Unclear | |  | | | | | |
|  | The investigator can imitate the inspiratory and expiratory state of pulmonary function examination to remind the respondents. | | | | | | | | |

End time of investigation (24-hour system)：□□h□□min

**Surveillance of chronic diseases and their risk factors in China（2013）**

**Body Measurement Record Form**

Personal code_：_□□□□□□□□□□

| **Height, weight, waist circumference and blood pressure enquiries** | | | |
| --- | --- | --- | --- |
| Hello, below we will ask you a few questions about your height, weight, waist circumference and blood pressure. | | | |
| K1 | Do you know your height? | 1 Yes，□□□.□centimeter（cm）  99 No | |
| K2 | Do you know your weight? | 1 Yes，□□□.□kilogram（kg）  99 No | |
| K3 | Do you know your waist circumference? | 1 Yes，□□□.□centimeter（cm）  99 No | |
| **Body measurements** | | | |
| Hello, next we will measure your height, weight, waist circumference and blood pressure, please cooperate. | | | |
| M1a | Name of Surveyor 1 | ________________________ | |
| M1b | Name of surveyor 2 | ________________________ | |
| M2 | Height  Note to investigators: record -9 if height is over the range. | □□□.□centimeter（cm） | |
| M3 | Weight  Note to investigators: record -9 if weight is over the range. | □□□.□kilogram（kg） | |
| **Waist Circumference** | | | |
| M4 | Waist Circumference | □□□.□centimeter（cm） | |
| **Blood pressure and heart rate** | |  | |
| M5 | Room temperature | □□.□ ℃ | |
| M6 | Name of Surveyor | ______________________________ | |
| M7a | 1st reading  Attention of investigator: Blood pressure is measured and recorded for the first time after the subject has rested for 5 minutes, and blood pressure and heart rate are measured for the second time after 1 minute of rest | Systolic pressure | □□□ mmHg |
| M7b |  | Diastolic blood pressure | □□□ mmHg |
|  |  | Heart rate | □□□times/minute |
| M7c |  |  |  |
| M8a | 2nd reading  Attention of investigator: Record the 2nd measurement and take the 3rd measurement of blood pressure and heart rate after the subject has rested for 1 minute. | Systolic pressure | □□□ mmHg |
| M8b |  | Diastolic blood pressure | □□□ mmHg |
| M8c |  | Heart rate | □□□times/minute |
| M9a | 3rd reading  Record the 3rd measurement | Systolic pressure | □□□ mmHg |
| M9b |  | Diastolic blood pressure | □□□ mmHg |
| M9c |  | Heart rate | □□□times/minute |
